# The Expression and Correlation of iNOS and p53 in Oral Squamous Cell Carcinoma

**DOI:** 10.1155/2015/637853

**Published:** 2015-10-07

**Authors:** Lan Yang, Youyuan Wang, Lvhua Guo, Liping Wang, Weiliang Chen, Bin Shi

**Affiliations:** ^1^The State Key Laboratory Breeding Base of Basic Science of Stomatology (Hubei-MOST) and Key Laboratory of Oral Biomedicine Ministry of Education, School and Hospital of Stomatology, Wuhan University, 237 Luoyu Road, Wuhan 430079, China; ^2^Key Laboratory of Oral Medicine, Guangzhou Institute of Oral Disease, Stomatology Hospital of Guangzhou Medical University, Guangzhou 510140, China; ^3^The Sun Yat-sen Memorial Hospital of Sun Yat-sen University, Guangzhou, Guangdong 510120, China

## Abstract

Oral squamous cell carcinoma (OSCC) is the most prevalent form of oral cancer. Inducible nitric oxide synthase (iNOS) and p53 are associated with a variety of human cancers, but their expression and interaction in OSCC have not been fully explored. In this study, we investigated the expression of iNOS and p53 in OSCC and their correlation with tumor development and prognosis. In addition, we explored the interaction of iNOS and p53 in OSCC. The expression of iNOS and p53 in OSCC was investigated using immunohistochemical method and their interaction was studied using RNAi technique. Our results showed that the expression of both iNOS and p53 was significantly correlated with tumor stages and pathological grade of OSCC (*P* < 0.05). In contrast, there was no correlation between iNOS and p53 expression and lymph node metastasis (*P* < 0.05). The OSCC survival rate was negatively associated with iNOS expression, but not with p53. A significant increase in the expression of the p53 was observed when iNOS expression was knocked down. The immunoexpression of iNOS is correlated with tumorigenesis and prognosis of OSCC and may serve as a prognostic marker.

## 1. Introduction

Nitric oxide (NO) is a small gas molecule involved in a variety of biological processes including neurotransmitter, vasodilation, and immune responses [1]. NO is believed to play roles in multiple stages of various cancers. It is involved in both promoting and inhibiting the etiology of cancer depending on tumor type, NO concentration, and tumor cell sensitivity to NO [2, 3].

Endogenous NO is synthesized from L-arginine by isoforms of nitric oxide synthase (NOS). Among the three isoforms of NOS, specifically, endothelial (eNOS), neuronal (nNOS), and inducible (iNOS), iNOS produces the greatest amount of NO and is the synthase isoform most commonly associated with malignant disease [4]. iNOS expression has been detected in both normal mammalian tissues and a wide range of human cancers such as neuroblastoma, colon adenocarcinoma, ovarian cancer, stomach cancer, liver cancer, breast cancer, and head and neck cancer [5–9]. Recently, iNOS overexpression was observed in oral squamous cell carcinoma (OSCC) [10]. However, the correlation between iNOS levels and tumor stages, differentiation grade, and survival rate has not been well explored in OSCC. Sappayatosok et al. reported that iNOS shows correlation with cervical lymph node metastasis and tumor staging in OSCC [11]. A positive correlation between iNOS mRNA n and neck lymph node metastasis was also reported in squamous cell carcinoma of tongue [12].

p53 is a product of TP53 gene and has been called “the guardian of the genome.” It was reported that p53 has a number of roles, in particular regulating cell cycle by halting the G1/S regulation point if DNA damage is present [13]. p53 is associated with a variety of human cancers [14]. In its wild-type form, p53 is a major tumor suppressor whose function is critical for protection against cancer [15]. In contrast, the mutant p53 protein (mt-p53) loses its original tumor suppressor and becomes a tumor-promoting factor and promotes the process of tumor [16–18]. High frequency of mutation in TP53 gene has been shown in a variety of human tumors such as breast, brain, rectum, colon, esophagus, and lung cancers and OSCC [19]. However, the relationship between p53 expression in OSCC and tumor stages has not been well studied.

The interactions between iNOS and p53 are complicated and still unclear. As the two genes most closely associated with the tumor, the expression of iNOS and p53 and the relationship in the process of tumor development has been the focus of attention. The majority of previous studies about iNOS and p53 focused on the expression and mutation of p53 [20]. The relation between iNOS and p53 has not been studied by decreasing iNOS expression using siRNA technique.

In this study, we first investigated the expression of iNOS and p53 in 16 cases of normal oral mucosa and 72 cases of OSCC. We then evaluated the correlation between their expression levels and tumor stages, differentiation grade, and survival rate. Furthermore, the expression of iNOS was knocked down using siRNA transfection. The role of iNOS on p53 expression and tumor cell growth was then investigated.

## 2. Material and Methods

### 2.1. Tissue Preparation

A total of 88 cases of surgical biopsies specimens were obtained from Sun Yat-sen University, China, which include 16 cases of normal oral mucosa and 72 cases of oral squamous cell carcinoma. Histologically, 50 cases were well differentiated, while 10 cases were of moderate differentiation and 12 cases of poor differentiation. There are 56 cases of OSCC in early I-II stage, 16 cases in III-IV stage, and 23 cases associated with regional lymph node metastasis. All of the patients did not receive any radiation treatment. The specimens were cut into 4 *μ*m thick serial sections, fixed in 10% formalin, and embedded in paraffin.

### 2.2. Reagents

Mouse anti-human iNOS monoclonal antibody and mouse anti-human p53 monoclonal antibody DO-7 were purchased from NeoMarkers (USA). Immunohistochemical detection kit was purchased from Dako (Denmark). Anti-rabbit-IgG-HRP and anti-mouse-IgG-HRP were purchased from Cell Signaling Technology (CST, USA).

### 2.3. Immunohistochemical Assay for Tissues

The streptavidin-biotin standard protocol was performed. Briefly, the tissue sections were deparaffinized in xylene and rehydrated in graded alcohol. Antigen retrieval was performed in citrate buffer using microwave method followed by incubation in 3% H_2_O_2_ at room temperature for 10 minutes to quench endogenous peroxidase. The sections were then incubated in blocking solution (3% bovine serum albumin) for 1 hour at room temperature, followed by incubation with iNOS and p53 primary antibodies overnight at 4°C. The secondary antibody was added in the next day. The tissues were then incubated for 30 min at room temperature and washed with PBS. Prior to microscopic examination, the sections were developed in DAB Chromogen and counterstained in Mayer's hematoxylin. The number of positive cells on each slice was counted based on 10 fields of view at ×200. The positive rate was calculated as the proportion of the total number of cells in the visual field. The results were divided into four levels according to the positive rate: <10% of positive cells were marked as negative (−); 10%–40% were weakly positive (+) and >50% were strongly positive (++).

### 2.4. Survival Analysis

The hospital visit and follow-up records were checked. The survival curves were plotted by Kaplan-Meier method and the survival rate was calculated by univariate analysis.

### 2.5. Tca8113 Cell in Culture

The Tca8113 cell lines were purchased from the Ninth People's Hospital of Shanghai, China. The Tca8113 cells of OSCC carry TP53 mutation. The cells were routinely cultured in DMEM low glucose medium containing 10% fetal bovine serum (FBS) and maintained in a 5% CO_2_ thermostatic cell incubator at 37°C.

### 2.6. iNOS Gene Silencing with siRNA

The siRNA-iNOS and negative controls were synthesized and purchased from Invitrogen, China. The iNOS gene sequences are iNOS siRNA1: 5′-ACAACAGGAACCUACCAGCTT-3′; 5′-GCUGGUAGGUUCCUGUUGUTT-3′; iNOS siRNA2: 5′-ACACAAGGCCAAUACCGACTT-3′; 5′-GCGUGUAUUGGCCUGUGUUTT′-3′; negative control siRNA: 5′-ACCAUAGGAUCCUACACGCTT-3′; 5′-GCGGCUAGCUUCCUUGUGUTT′-3′. siRNA was used at a concentration of 30 nM using Lipofectamine 2000 liposomes (Invitrogen) as transfection agent (0.02%). After 24 h, Tca8113 cells were transfected with siRNA according to the manufacture's protocol. The transfection of each group of cells was observed under a fluorescence microscope.

### 2.7. qRT-PCR Analysis

Gene expression was analyzed by quantitative real-time PCR (qRT-PCR) using SYBR Green PCR Master Mix. The following genes were evaluated: iNOS gene (forward primer: 5′-CAGCGGGATGACTTTCCAAG-3′; reverse primer: 5′-AGGCAAGATTTGGACCTGCA-3′); p53 gene (upstream primer: 5′-ACGGTGACACGCTTCCCTGGATTGG-3′, downstream primer: 5′-CTGTCAGTGGGGAACAAGAAGTGGAGA-3′). GAPDH gene (upstream primer 5′′-CCTGGACCACCCAGCCAGCAA-3′, downstream primer: 5′-TGTTATGGGGTCTGGGATA-3′). Four groups were set up including si-1, si-2, Si-NC (negative control), and MOCK (blank control). GAPDH was used as the loading control. The experiment was repeated four times.

### 2.8. Western Blot Analysis

The total protein was extracted using BCA method. Western blot analysis of iNOS and p53 protein expression was performed using primary antibodies including anti-iNOS (of NOS2) and p53 antibody and the corresponding secondary antibodies. GAPDH was used as the internal reference. The specific bands were detected using West Pico Chemiluminescent Substrate (Pierce, USA) and the blots were exposed onto the Hyperfilms. The band intensity was quantified by the AlphaImager 2200 software. The experiment was repeated 4 times.

### 2.9. Cell Viability Analysis

Cell viability analysis was performed using Cell Counting Kit-8 (CCK-8) according to the manufacture's protocol. Briefly, the transfected cells' suspension (100 *μ*L/well) was inoculated into a 96-well plate and preincubated in a humidified incubator (37°C, 5% CO_2_). When the cell density reached 50%, siRNA-iNOS was transfected into the cells. At 48 h, after transfection, the medium was removed from the wells and 10 *μ*L of CCK8 solution was added to each well. The plate was incubated for another 2 hours. The absorbance was measured at 450 nm using a microplate reader. Triplicates were set up for each group.

### 2.10. Statistics Analysis

SPSS16.0 software was used for statistical analysis (*α* = 0.05). The expression of iNOS and p53 and the correlation were analyzed by Pearson correlation analysis. The survival curves were plotted by Kaplan-Meier method and the survival rate was calculated by univariate analysis.

## 3. Results

### 3.1. The Distribution and Expression of iNOS and p53 Protein in Normal Oral Epithelium and OSCC

**Figure 1:**
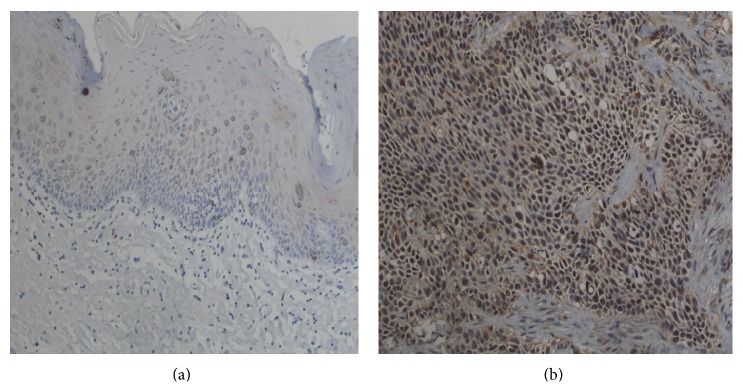
The expression of iNOS in normal and OSCC tissues. (a) Normal control and (b) OSCC tissues. Magnification: ×200.

**Figure 2:**
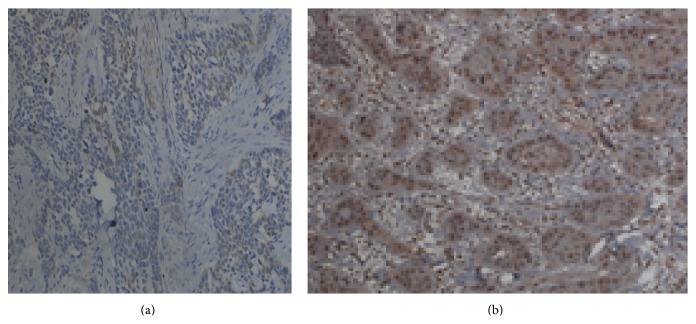
The expression of p53 protein in normal and OSCC tissues. (a) Normal control and (b) OSCC tissues. Magnification: ×200.

As shown in Figure 1, iNOS is mainly localized in the cytoplasm with brownish yellow as positive expression. p53 was clearly observed in the cytoplasm and the nucleus. The brown particles indicated the positive expression of p53 (Figure 2). The expression of iNOS and p53 in normal oral epithelium and OSCC was significantly different. Two cases out of 16 normal oral epithelium samples have positive iNOS expression. In contrast, iNOS showed positive expression in 42 out of 72 OSCC samples (Table 1). Similarly, positive p53 expression was only observed in 3 out of 16 normal oral epithelium samples compared to 42 out of 72 in OSCC samples. In addition, both proteins showed significant higher expression in oral squamous cell carcinoma than that in normal control groups (*P* < 0.05).

### 3.2. Correlation between iNOS and p53 Expressions in OSCC

Four-two cases (58.33%) of OSCC showed the positive expression of iNOS and 45 cases (62.50%) of OSCC have positive p53 expression (Table 2). The immunohistochemical expression of iNOS and p53 was positively correlated (*P* < 0.01).

### 3.3. The Immunoexpression of iNOS and p53 Was Associated with OSCC Stages and Pathologic Grade

OSCC was divided into four stages (I–IV) based on tumor size and nodal involvement. The prognosis of OSCC was also evaluated using histologic grade including moderate and poor differentiation. Our results showed that the expression of iNOS and p53 correlated significantly with the stages of OSCC (*P*_iNOS_ < 0.05, *P*_p53_ < 0.05) and pathologic differentiation grade (*P*_iNOS_ < 0.05, *P*_p53_ < 0.05). However, there was no correlation between lymph node metastasis and the immunoexpression of both iNOS and p53 (*P*_iNOS_ > 0.05, *P*_p53_ > 0.05) (Table 3). In addition, the pathological characteristics were not associated with the combined expression of both iNOS and p53 (*P*_iNOS+p53_ > 0.05).

### 3.4. Survival Analysis

**Figure 3:**
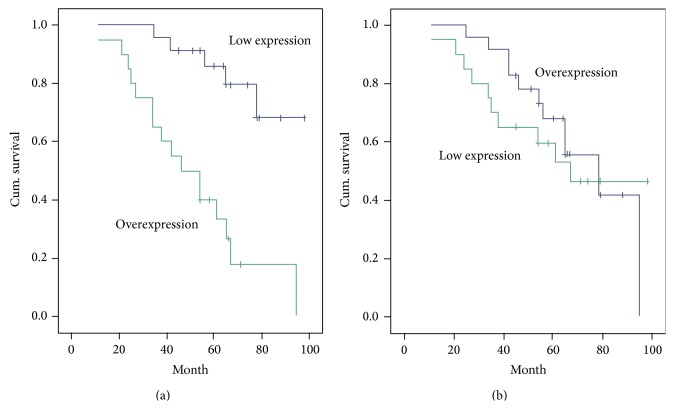
Survival analysis based on the expression of iNOS (a) and p53 (b).

Figure 3 showed the Kaplan-Meier survival estimates based on the expression of iNOS and p53. The overall survival of patients with positive iNOS expression at 5 years was 38.8%, whereas the overall survival of patients with negative iNOS expression was 80.1% (*P* < 0.01) (Figure 3(a)). However, there was no statistical difference in the survival rate between positive and negative p53 expression groups (*P* > 0.05).

### 3.5. The Interaction between iNOS and p53 Expressions

To further investigate the correlation between iNOS and p53 expressions in OSCC, we knocked down the expression of iNOS in OSCC Tca8113 cells through siRNA transfection.

**Figure 4:**
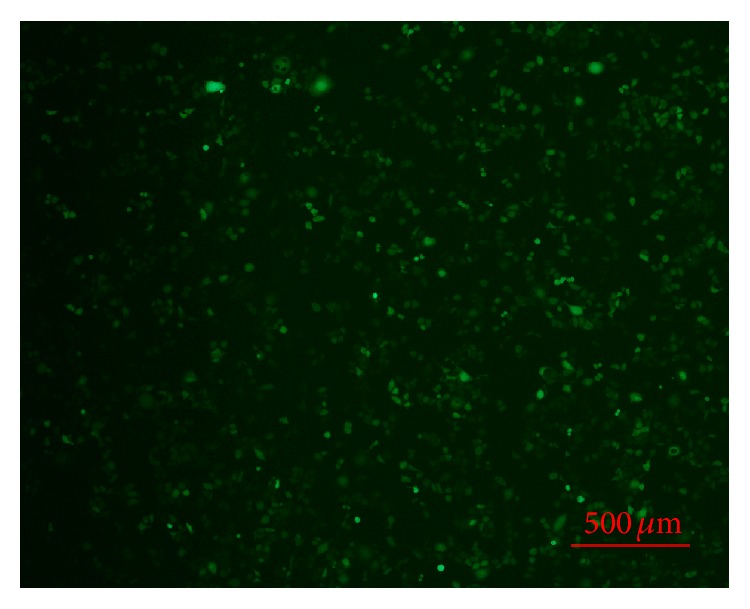
Tca8113 cells transfected with siRNA-INOS (si-1).

**Figure 5:**
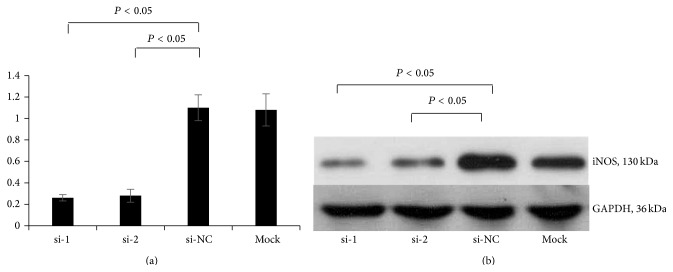
The expression of iNOS in Tca8113 cells transfected with iNOS-siRNA. (a) iNOS expression measured by RT-PCR; (b) iNOS expression measured by western blot. si-1: iNOS-siRNA-1; si-2: iNOS-siRNA-2; si-NC: negative control; si-MOCK: mock transfection of siRNA vector.

**Figure 6:**
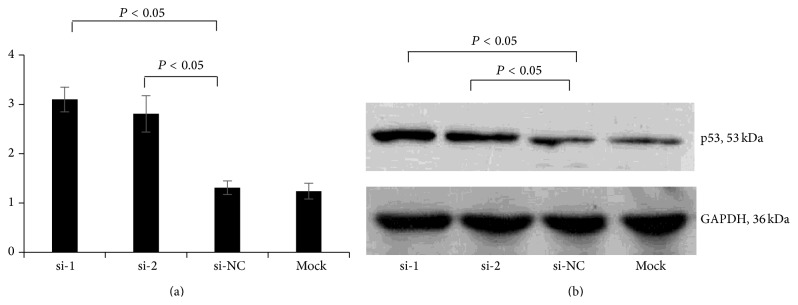
The expression of p53 in Tca8113 cells transfected with iNOS-siRNA. (a) p53 expression measured by RT-PCR; (b) p53 expression measured by western blot. si-1: iNOS-siRNA-1; si-2: iNOS-siRNA-2; si-NC: negative control; si-MOCK: mock transfection of siRNA vector.

Two siRNA sequences (si-1 and si-2) and a negative control (si-NC) of siRNA-iNOS were transfected into Tca8113 cells (Figure 4). The expression of iNOS and p53 after iNOS silencing was investigated using RT-PCR and western blot. The results showed that the expression of iNOS was effectively knocked down in si-1 and si-2 transfection groups (Figures 5(a) and 5(b)). However, the expression of p53 was significantly enhanced in response to the silencing of iNOS in Tca8113 cells (*P* < 0.05) (Figures 6(a) and 6(b)).

**Figure 7:**
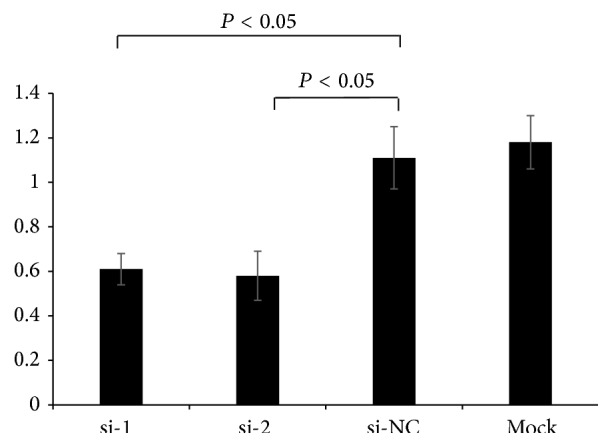
The effect of iNOS silencing on the proliferation of Tca8113 cells measured by CCK-8 method. si-1: iNOS-siRNA-1; si-2: iNOS-siRNA-2; si-NC: negative control; si-MOCK: mock transfection of siRNA vector.

Furthermore, the effect of iNOS silencing on Tca8113 cell proliferation was evaluated using Cell Counting Kit-8. Our results showed that the proliferation of Tca8113 cells was significantly inhibited when iNOS gene was silenced (*P* < 0.05) (Figure 7).

## 4. Discussion

iNOS is a key enzyme in the synthesis of endogenous NO. Increased iNOS expression has been shown in a number of carcinomas including OSCC, human gastric cancer, colitis, and colon cancer [10, 21]. In this study, we further demonstrated that the expression of iNOS is correlated with tumor grade and proliferation rate in OSCC.

p53 plays an important role in apoptosis, genomic stability, and inhibition of angiogenesis and thus functions as a key tumor suppressor. However, mutant p53 proteins gain oncogenic properties favoring the insurgence, the maintenance, and the spreading of malignant tumors [22]. More than 60% of our OSCC samples were p53 positive which is consistent with the results described in the literature [23]. The detection of p53 expression in a large percentage of OSCC cases by immunohistochemistry indicated the altered status of this protein [24]. Previous reports are inconclusive in terms of the relation between p53 immunoexpression and the differentiation grade of OSCC [24–26]. Here, our results clearly showed that p53 expression is significantly correlated with the tumor stages and differentiation grade of OSCC, but not with lymph node metastasis.

In this study, we demonstrated for the first time that 5-year survival time of patients with positive iNOS expression was dramatically shorter than that of those with negative iNOS expression in OSCC. In contrast, the expression of p53 has no effect on 5-year survival time of patients with OSCC. Similarly, there was no difference in rates for overall and disease-free survival between patients with p53-positive and -negative head and neck squamous cell carcinoma [27]. However, p53 was reported to be predictive of a poor prognosis in squamous cell carcinoma of the uterine cervix [28]. It is probably due to the mutation of p53 protein in the late stages of squamous cell carcinoma.

The interaction of NO, iNOS, and p53 in tumor growth and angiogenesis is complicated and still unclear. Davis et al. reported that the activation of p53 is dependent upon iNOS expression in lung cancer [29]. iNOS is associated with the mutation of p53 in human esophageal squamous cell carcinoma [30]. To study the role of iNOS, the majority of previous studies overexpressed the iNOS isoform which could result in nonphysiological extremely high levels of NO. In this study, we also investigated the role of iNOS on tumor growth and p53 expression by decreasing endogenous iNOS expression using siRNA technique. The siRNA transfected Tca8113 cells displayed significantly reduced growth and increase in p53 expression. Using a similar approach, Kostourou et al. reported that decreased iNOS expression resulted in the reduction of C6 cell growth [31]. Previous studies showed that silencing of mutated p53 led to the decrease of the growth of human lung adenocarcinoma cells [32]. There is still no report related to the expression of iNOS in response to the silencing of mutated p53, which will be our future objective.

All the above evidences indicated that iNOS and p53 have complex interaction in the process of tumor development. The interaction is not only related to the changes of gene and protein expression, but also to the changes of the nature and function of p53, such as activation and mutations. In the progression of OSCC tumor tissues, p53 expression is increased with the increased expression of iNOS because part of the p53 mutated and become mt-p53, which makes it a tumor-promoting factor. When the expression of iNOS was reduced in Tca8113 cells by RNA interference technology, the local NO concentration was reduced which promoted the increased expression of wt-p53 and thus led to the inhibition of tumor cell growth.

## 5. Conclusion

In conclusion, we have shown that (1) iNOS and p53 positivity were observed in 58.33% and 62.50% of OSCC, respectively. (2) The expression of iNOS and p53 was associated with OSCC and pathologic grade but did not correlate with lymph node metastasis. (3) The OSCC survival rate was negatively associated with iNOS expression, but not p53. (4) The interaction between p53 and iNOS is complicated. iNOS and p53 expression was positively correlated in the progression of OSCC tumor tissues. In the tumor cells, p53 expression was upregulated in response to iNOS silencing. Together, these data further support the concept of inhibiting iNOS as a therapeutic strategy for the treatment of OSCC.

## Figures and Tables

**Table 1:** iNOS and p53 expression in different tissues.

Groups	Case number (*n*)	iNOS		*P*	p53		*P*
		−	+ (%)		−	+ (%)	
Normal	16	14	2 (12.5%)	<0.05	13	3 (18.75%)	<0.05
OSCC	72	30	42 (58.33%)		27	45 (62.50%)	

**Table 2:** The correlation between iNOS and p53 expressions in OSCC.

		p53		Total (%)
		−	+	
iNOS	−	25	5	30
	+	2	40	42 (58.33%)

	Total (%)	27	45 (62.5%)	
		*χ* ^2^ = 54.10	*P* < 0.01	

**Table 3:** The correlation between iNOS and p53 protein expressions and pathologic grade.

Groups/classification	Case number (*n*)	iNOS		*P* value	p53		*P* value
		−	+ (%)		−	+	
Tumor stage							
I~II stage	56	27	29 (51.79%)	*P* < 0.05	26	30 (53.18%)	*P* < 0.05
III~IV stage	16	3	13 (81.25%)		1	15 (93.75%)	
Pathologic differentiation grade							
Moderate	60	28	32 (53.33%)	*P* < 0.05	26	34 (56.67%)	*P* < 0.05
Poor	12	2	10 (83.33%)		2	12 (91.67%)	
Lymph node metastasis							
Have	23	10	13 (56.52%)	*P* > 0.05	9	14 (60.87%)	*P* > 0.05
No	49	20	29 (59.18%)		18	31 (63.27%)	

## References

[1] Schmidt H. H. H. W., Walter U. (1994). NO at work. *Cell*.

[2] Xu W., Liu L. Z., Loizidou M., Ahmed M., Charles I. G. (2002). The role of nitric oxide in cancer. *Cell Research*.

[3] Choudhari S. K., Chaudhary M., Bagde S., Gadbail A. R., Joshi V. (2013). Nitric oxide and cancer: a review. *World Journal of Surgical Oncology*.

[4] Lechner M., Lirk P., Rieder J. (2005). Inducible nitric oxide synthase (iNOS) in tumor biology: the two sides of the same coin. *Seminars in Cancer Biology*.

[5] Brennan P. A., Dennis S., Poller D., Quintero M., Puxeddu R., Thomas G. J. (2008). Inducible nitric oxide synthase: correlation with extracapsular spread and enhancement of tumor cell invasion in head and neck squamous cell carcinoma. *Head & Neck*.

[6] Vakkala M., Kahlos K., Lakari E., Pääkkö P., Kinnula V., Soini Y. (2000). Inducible nitric oxide synthase expression, apoptosis, and angiogenesis in in situ and invasive breast carcinomas. *Clinical Cancer Research*.

[7] Brennan P. A., Sharma S., Bowden J. R., Umar T. (2003). Expression of inducible nitric oxide sythase in bone metastases. *European Journal of Surgical Oncology*.

[8] Broholm H., Rubin I., Kruse A. (2003). Nitric oxide synthase expression and enzymatic activity in human brain tumors. *Clinical Neuropathology*.

[9] Speranza L., De Lutiis M. A., Shaik Y. B. (2007). Localization and activity of iNOS in normal human lung tissue and lung cancer tissue. *International Journal of Biological Markers*.

[10] Connelly S. T., Macabeo-Ong M., Dekker N., Jordan R. C. K., Schmidt B. L. (2005). Increased nitric oxide levels and iNOS over-expression in oral squamous cell carcinoma. *Oral Oncology*.

[11] Sappayatosok K., Maneerat Y., Swasdison S. (2009). Expression of pro-inflammatory protein, iNOS, VEGF and COX-2 in oral squamous cell carcinoma (OSCC), relationship with angiogenesis and their clinico-pathological correlation. *Medicina Oral, Patologia Oral y Cirugia Bucal*.

[12] Chen W.-L., Zeng S.-G., Li H.-G., Huang H.-Z., Pan C.-B. (2002). Expression of inducible nitric oxide synthase mRNA in squamous cell carcinoma of tongue. *Ai Zheng*.

[13] Giono L. E., Manfredi J. J. (2006). The p53 tumor suppressor participates in multiple cell cycle checkpoints. *Journal of Cellular Physiology*.

[14] Bieging K. T., Mello S. S., Attardi L. D. (2014). Unravelling mechanisms of p53-mediated tumour suppression. *Nature Reviews Cancer*.

[15] Mercer W. E. (1992). Cell cycle regulation and the p53 tumor suppressor protein. *Critical Reviews in Eukaryotic Gene Expression*.

[16] Weisz L., Damalas A., Liontos M. (2007). Mutant p53 enhances nuclear factor *κ*B activation by tumor necrosis factor *α* in cancer cells. *Cancer Research*.

[17] Zalcenstein A., Weisz L., Stambolsky P., Bar J., Rotter V., Oren M. (2006). Repression of the MSP/MST-1 gene contributes to the antiapoptotic gain of function of mutant p53. *Oncogene*.

[18] Liu D. P., Song H., Xu Y. (2010). A common gain of function of p53 cancer mutants in inducing genetic instability. *Oncogene*.

[19] Strano S., Dell'Orso S., Di Agostino S., Fontemaggi G., Sacchi A., Blandino G. (2007). Mutant p53: an oncogenic transcription factor. *Oncogene*.

[20] Leroy B., Girard L., Hollestelle A., Minna J. D., Gazdar A. F., Soussi T. (2014). Analysis of TP53 mutation status in human cancer cell lines: a reassessment. *Human Mutation*.

[21] Gochman E., Mahajna J., Shenzer P. (2012). The expression of iNOS and nitrotyrosine in colitis and colon cancer in humans. *Acta Histochemica*.

[22] Strano S., Dell'Orso S., Mongiovi A. M. (2007). Mutant p53 proteins: between loss and gain of function. *Head and Neck*.

[23] Abrahao A. C., Bonelli B. V., Nunes F. D., Dias E. P., Cabral M. G. (2011). Immunohistochemical expression of p53, p16 and hTERT in oral squamous cell carcinoma and potentially malignant disorders. *Brazilian Oral Research*.

[24] Gasco M., Crook T. (2003). The p53 network in head and neck cancer. *Oral Oncology*.

[25] de Oliveira L. R., Ribeiro-Silva A., Zucoloto S. (2007). Prognostic impact of p53 and p63 immunoexpression in oral squamous cell carcinoma. *Journal of Oral Pathology and Medicine*.

[26] Abrahao A. C., Bonelli B. V., Nunes F. D., Dias E. P., Cabral M. G. (2011). Immunohistochemical expression of p53, p16 and hTERT in oral squamous cell carcinoma and potentially malignant disorders. *Brazilian Oral Research*.

[27] Oridate N., Homma A., Higuchi E. (2009). p53 expression in concurrent chemoradiotherapy with docetaxel for head and neck squamous cell carcinoma. *Auris Nasus Larynx*.

[28] Moon A., Won K. Y., Lee J. Y., Kang I., Lee S.-K. (2011). Expression of BDNF, TrkB, and p53 in early-stage squamous cell carcinoma of the uterine cervix. *Pathology*.

[29] Davis D. W., Weidner D. A., Holian A., McConkey D. J. (2000). Nitric oxide-dependent activation of p53 suppresses bleomycin-induced apoptosis in the lung. *Journal of Experimental Medicine*.

[30] Matsumoto M., Furihata M., Kurabayashi A., Araki K., Sasaguri S., Ohtsuki Y. (2003). Association between inducible nitric oxide synthase expression and p53 status in human esophageal squamous cell carcinoma. *Oncology*.

[31] Kostourou V., Cartwright J. E., Johnstone A. P. (2011). The role of tumour-derived iNOS in tumour progression and angiogenesis. *British Journal of Cancer*.

[32] Ma L. L., Sun W. J., Wang Z., Zh G. Y., Li P., Fu S. B. (2006). Effects of silencing of mutant p53 gene in human lung adenocarcinoma cell line Anip973. *Journal of Experimental and Clinical Cancer Research*.

